# Effectiveness of Gatekeepers in Determining the Appropriate Use of Brain MRI/MRA Tests

**DOI:** 10.1155/2014/670915

**Published:** 2014-05-26

**Authors:** Seiji Bito, Shinji Matsumura, Kazuhiko Kotani, Shunichi Fukuhara

**Affiliations:** ^1^Division of Clinical Epidemiology, National Hospital Organization Tokyo Medical Center, 2-5-1 Higashigaoka, Meguro-ku, Tokyo 152-8602, Japan; ^2^Department of Clinical Laboratory Medicine, Jichi Medical University, 3311-1 Yakushiji, Shimotsuke-shi, Tochigi-ken 329-0498, Japan; ^3^Department of Epidemiology and Healthcare Research, Kyoto University Graduate School of Medicine, Yoshida-Konoe-cho, Sakyo-ku, Kyoto 606-8501, Japan

## Abstract

The purpose of the study is to examine whether, among patients who visited hospitals and underwent brain MRI or MRA scan tests, there was a relationship between the existence of clinically significant abnormal findings and the relevance of primary care physicians' referrals. A case-control study was carried out at six teaching hospitals in Japan. We identified cases with significant abnormal MRI/MRA findings from radiologists' reports based on certain explicit criteria and controls with outpatients who underwent MRI/MRA scans but did not have stroke. We also collected clinical data independently from medical records. The findings of 156 cases and 721 controls were collected for the analysis. A multivariate analysis adjusted by age group, sex, and the number of comorbidity factors showed that those who had visited the hospitals after referral were more likely to have significant abnormal findings in their MRI/MRA scan results (odds ratio [OR] = 1.6, 95% CI: 1.1 to 2.4). The present study suggests that referral from gatekeepers such as primary care physicians is effective in determining the appropriate use of brain MRI/MRA tests for hospital outpatients.

## 1. Introduction

In health insurance payment systems with a fee-for-service system, insurance expenses are paid to health care providers according to the number of tests or the amount of treatment. As a result, the problem of necessary medical services not being provided can be prevented; however, the problem of overuse of medical services arises, such as expensive tests being performed on patients with very low pretest probability [1–4]. The Japanese health insurance system for outpatient services implements universal care based on a social insurance system, which utilizes a fee-for-service system [5]. Under this system, citizens have free access to visit hospitals and encounter very few barriers when they undergo necessary tests or treatment [6, 7]. However, consequently, there are over 10,000 units of CT equipment and approximately 6,000 units of MRI equipment in Japan, and, hence, the economic burden on patients due to the overuse of expensive testing equipment has increased [8, 9]. Whereas the cost for no contrast brain MRI scan, which is approximately 1,500 bucks in the United States, is less expensive in Japan, it is approximately 400 bucks.

Against such a background, in order to receive appropriate medical services, the importance of the existence of “primary care physicians” has recently been under focus [10, 11]. If the contribution of “primary care physicians” as in restricting overprescription is proven in Japan, then the meaning of “primary care physicians” as gatekeepers will become clear [12–14]. With regard to the effect of primary care physicians in restricting overprescription, there have been several reports in Europe and the United States, particularly regarding treatment-related matters [15, 16]. On the other hand, there is no clear understanding as to whether primary care physicians prevent overuse of expensive diagnostic equipment associated with diagnoses of symptoms and signs.

In this evaluation, we conducted a case-control study among consecutive patients who visited general hospitals equipped with MRI scanner and underwent head MRI or MRA tests for their complaints during observation period, to examine whether or not there was a relationship between the existence of clinically significant abnormal findings and patients who were referred from primary care physicians. By clarifying the meaning of primary care physicians as gatekeepers in the current situation, their function in the insurance system can be more positively viewed.

## 2. Materials and Methods

A case-control study was adopted for the research design. In a total of six general hospitals that carry MRI diagnostic equipment, a case group and a control group were sampled, and, subsequently, a research study was conducted on the results of the interpretation by radiologists and other clinical data in the medical records. Those studied were confined to outpatients with major neurological complaints such as headaches, dizziness, staggering, fainting, and temporary unconsciousness disorder who underwent head MRI or MRA tests at the above-mentioned hospitals during the 18-month period from February 2004 to July 2005. We excluded the patients who had any of the following conditions from the study: those who underwent head MRI or MRA tests due to similar major complaints 60 days prior to undergoing head MRI or MRA tests retroactively, those who had already undergone diagnoses for brain tumors or cerebrovascular damage 60 days prior to undergoing head MRI or MRA tests retroactively, those who underwent diagnoses for head injuries 60 days prior to undergoing head MRI or MRA tests retroactively, those who had a previous clinical history of malignant tumors, and emergency room patients. The case group included patients who had clinically significant intracranial neoplastic lesions or ischemic or hemorrhagic lesions, and the control group included patients who did not have these findings. The established criteria are shown in Table 1.

As for potential explanatory variables associated with outcomes, that is, positive findings on MRI, patient characteristics (age, sex, complications, previous diseases, and smoking history), diagnosis at the first visit to the hospital, and the existence of referrals from primary care physicians leading to head MRI or MRA tests were all collected. All clinical information was collected from medical records and reports from the radiology departments.

In collecting data, firstly, a list of all of the patients who underwent head MRI or MRA tests at the above-mentioned hospitals cooperating with the study during the 18-month period from February 2004 to July 2005 was created. The information on the list included only date of test, medical record number, and sex. Secondly, previous clinical history and major complaints that led to tests were determined as identification standards, based on the medical records of the patients or the order input screen, and the patients on the patient list were divided into patients to be studied and patients not to be studied. Statistical reference numbers (arbitrary 4-digit numbers) were written down beside the medical record number of the patients to be studied. This patient list showed the codes of the patients to be studied, and the statistical reference numbers were anonymously linkable. Based on the medical records or order input screen and reports from the radiology departments, the person in charge of the study at each hospital filled in data in the registration forms for the patients, and the researchers classified the patients (to be studied) either as (belonging to) case groups or as control group.

When collecting clinical information, explanations to and acquisition of written consent from the patients were not carried out for individual patients; however, the details of the study were posted in the hospitals implementing the study. Data collected at each hospital was accumulated at the Division on Clinical Epidemiology, Tokyo Medical Center, as anonymously linkable information, and analyzed as a single batch. Regarding the relationship between the endpoints and explanatory variables, univariate analysis was conducted by an* X* ^2^-test, and logistic regression analysis was conducted with adjustment for age group, sex, the number of comorbidities (hypertension, diabetes, and hyperlipemia), and existence of a smoking habit. Adjusted odds ratio was calculated for displaying likelihood on the dependent variable. A level of significance was set as *P* < 0.05. SPSS version 13 was used for the analysis.

## 3. Results and Discussion

### 3.1. Results

From six hospitals in total, the available data from a total of 877 cases, represented by 156 cases in the case group and 721 cases in the control group, was collected. With regard to the tests, patients who underwent both head MRI and MRA accounted for 33% of all patients. Female patients accounted for 59% of all patients, and, as for age groups, 49 years old and younger accounted for 23%, 50–64 years old accounted for 38%, and 65 years old and older accounted for 38%.

The distribution of the major complaints of the registered patients, the distribution of smoking history, sex, and age groups in the case group and the control group, and the details of the disorders of both the case and the control groups are shown in Table 2. The average age and the standard deviation were 65 ± 16 years and 60 ± 18 years in the case group and control group, respectively. With regard to the comorbidities, the ratios of comorbidity with hypertension were 31% in the case group and 21% in the control group, the ratios of comorbidity with diabetes were 14% and 8%, the ratios of comorbidity with hyperlipemia were 18% and 15%, and the ratios of patients with one of the three comorbidities were 41% and 32%, respectively. Regarding smoking, the ratios of patients currently with a smoking habit in the case group and control group were 8% and 13%, respectively.

In Table 2, a comparison regarding the frequency of referrals from primary care physicians for all of the patients and the patients who visited the hospitals complaining of headaches, in the case group and control group, is shown. While patients with referrals from primary care physicians, among the patients with clinically significant findings of MRI/MRA tests, accounted for 39%, patients with referrals from primary care physicians in the patient group without clinically significant findings of MRI tests accounted for 27% (odds ratio: 1.8 and 95% confidence interval: 1.2–2.5). Furthermore, among 248 patients who visited the hospitals with a chief complaint of headache, the percentages of those with referrals from primary care physicians were 39% and 25% (odds ratio: 1.9 and 95% confidence interval: 0.9–4.2) in the case group and control group, respectively.

Table 3 shows the results of a logistic regression analysis by sex and age group, with the existence of a smoking history as a regulating factor, as well as the results of an analysis with clinically significant findings of MRI/MRA tests as outcomes. The results showed that having referrals from primary care physicians is significantly related to the outcomes (odds ratio: 1.6 and 95% confidence interval: 1.1–2.4). Moreover, based on a subgroup analysis limited to the group of patients who visited the hospitals with headache as a chief complaint, a similar tendency was also observed in the relationship between the explanatory factors and the outcomes; however, a statistically significant difference was not observed (odds ratio: 1.9 and 95% confidence interval: 0.8–4.4).

### 3.2. Discussion

The results of this study suggest that patients who visit their primary care physicians with some sort of neurological symptoms can avoid undergoing unnecessary MRI/MRA because of these primary care physicians. The results that we present include some limitations. In general, in case-control studies, in which data is collected based on previous medical records, other confounding factors related to the outcome have no small influence on describing the relationship between causative variables and the intended outcome. Furthermore, when these confounding factors are added to an analysis model after being measurable, these data usually cannot be sufficiently collected from previous records. In this study, while the severity of the patients' subjective symptoms and the duration of the symptoms and some risk factors such as diabetes are also assumed to be confounding factors that influence the existence or nonexistence of clinically significant abnormal findings of MRI/MRA, which were defined as the outcome, sufficient data could not be collected for adding to the analysis model in the analysis, for associated factors other than basic characteristics distribution, such as age and sex. Moreover, the findings of MRI/MRA, which are the outcome, are essentially parameterized based on reports of the diagnostician who interpreted the radiogram, and, hence, the adequacy and reliability are limited. However, with regard to patient sampling, outcome measurement, and the existence of referrals from primary care physicians, which is an explanatory variable, data was independently collected, and, therefore, no arbitrariness that would lead to an overinterpretation of the results occurred in the process of collecting data. In addition, regarding the severity of the patients' symptoms, which was considered to be the confounding factor that most affects the outcome, it is conceptually possible that when the symptoms are more severe, the patients would directly visit the hospitals in Japan, and, thus, patients' disease severity can be the confounding factor by indication (of seeing PC physician first) and this would generally underestimate the hypothesized association. In this sense, presence of some confounders, if any, would bias the results away from the hypothesized function of primary care physicians as gatekeepers. This tendency supports the fact that our results have little risk of alfa errors and even our nonsignificant results contain beta errors.

Headaches, dizziness, staggering, numbness, and so forth are very common symptoms that everyone has experienced and normally do not involve causative disorders with obvious brain abnormalities. On the other hand, it is difficult to deny that these symptoms are caused by critical disorders with a relatively high prevalence rate in Japanese, and which have a large impact on prognosis, such as brain infarctions. For those receiving medical care, the appearance of these symptoms induces considerable anxiety, and, hence, the assessments of medical professionals are necessary. In outpatient clinics of general hospitals in Japan, assessments by tests such as MRI have to be concentrated upon due to time constraints and so forth. However, conducting expensive tests gratuitously is not a desirable choice for patients or in terms of medical economics [17, 18]. If a primary assessment is provided at a clinic and so forth and a determination whether a thorough examination is necessary at the level of primary care, these events, which cause concern, can be avoided.

According to our study results, it was clarified that referrals from primary care physicians are related to higher proportion of those with clinically significant MRI findings. It is probable that the medical staff of large hospitals select MRI tests for patients who visit the hospitals directly as a primary assessment without thorough history gathering and physical examination, consequently resulting in overuse of MRI tests. It is believed that a screening function will work for very common symptoms, such as tension-type headaches, through primary care physicians [12], and, consequently, effective medical services can be provided. Our results are therefore expected to be a basis for proving this conceptual speculation.

## 4. Conclusion

A significantly higher proportion of clinically abnormal findings was observed in patients who were referred from primary care physicians and underwent head MRI/MRA tests, compared to patients who visited hospitals directly and underwent MRI/MRA tests. This supports our hypothesis that primary care physicians play an important role in functioning as gatekeepers. The roles and functions of primary care physicians in Japanese primary care include the continuity of medical care and dealing with the wide range of the medical care system, and, therefore, it is necessary in the future to further validate the effects of these roles and functions.

## Figures and Tables

**Table 1:** Criteria for defining the case group and control group.

		Diagnostic names of medical care information system (major complaints in undergoing tests)	Diagnostic names obtained from the results of radiogram interpretations
Case group (*n* = 156)	Criteria of case 1	(i) Headaches (ii) Dizziness (iii) Staggering (iv) Fainting (v) Temporary consciousness disorder (vi) Hyposthenia, palsy, dysmobility (vii) Peripheral nerve symptoms (viii) Brain tumors, possible brain tumors (ix) Cerebrovascular damage, possible cerebrovascular damage	Patients who were diagnosed as having brain tumors in the results of interpretations of radiograms of head MRI tests.
	Criteria of case 2		Patients who had clinically significant cerebrovascular damage (stenosis and cerebral stroke) identified in the results of interpretations of radiograms of head MRA or MRI tests^Note 1,2^.
Control group (*n* = 721)	Criteria of control	Applying to any of the above names (more than one item can be selected)	Patients who did not show clinically significant abnormalities upon head MRI or head MRA tests.

Note 1: definition of clinically significant cerebrovascular stenosis.

Stenosis in 50% of the internal carotid artery, common carotid artery, forebrain/midbrain/hindbrain artery, vertebral artery, and basilar artery.

Note 2: definition of clinically significant cerebral stroke.

(i) Findings of brain infarction on images that clearly explain symptoms in the medical records.

(ii) Findings of brain infarction in diffusion MRI.

(iii) Findings of brain hemorrhaging determined to be within four weeks after onset.

**Table 2:** List of character distributions in the case group and control group.

	Case group (*n* = 156)	Control group (*n* = 721)
Age (average ± SD)	65 ± 16	60 ± 18
Comorbidity (%)		
Hypertension	31	21
Diabetes	14	8
Hyperlipemia	18	15
At least one comorbidity	41	32
Existence of smoking habit (%)	8	13
Existence of referrals from primary care doctors (%)	39	27

**Table 3:** Relationship between referrals from primary care doctors and significant abnormal findings upon MRI: results of logistic regression analysis.

	Odds ratio [95% confidence interval]	
	All samples (*N* = 868)^Note 3^	Only patients with headaches as a chief complaint (*N* = 247)^Note 4^
Females (against males)	0.5 [0.3–0.7]	0.6 [0.3–1.4]
70 years old and older (compared to 19 years old and younger)	1.5 [1.0–2.1]	1.1 [0.5–2.7]
Existence of a smoking habit (compared to nonexistence)	0.4 [0.2–0.8]	0.2 [0.02–1.5]
The number of addictions (compared to no addiction)	1.3 [1.1–1.6]	1.3 [0.9–2.1]
Existence of referrals from primary care doctors (compared to nonexistence)	1.6 [1.1–2.4]	1.9 [0.8–4.4]

Note 3: nine of all 877 samples dropped out of the analysis because of missing data in the model variables.

Note 4: one of all 248 samples dropped out of the analysis because of missing data in the model variables.
